# A novel organ preservation solution with efficient clearance of red blood cells improves kidney transplantation in a canine model

**DOI:** 10.1186/s13578-018-0226-2

**Published:** 2018-04-10

**Authors:** Sheng Wang, Kristyn Gumpper, Tao Tan, Xianzhang Luo, Hui Guo, Changsheng Ming, Hanying Jiang, Jiangguo Fang, Guang Du, Hua Zhu, Jianjie Ma, Zhishui Chen, Nianqiao Gong

**Affiliations:** 1 Institute of Organ Transplantation, Key Laboratory of the Ministry of Health and the Ministry of Education, Tongji Hospital, Tongji Medical College, Huazhong University of Science and Technology, Wuhan, 430030 China; 2 Department of Surgery, Davis Heart and Lung Research Institute, The Ohio State University, Columbus, OH 43210 USA; 3 Department of Molecular and Cellular Biochemistry, The Ohio State University, Columbus, OH 43210 USA; 4 Department of Pharmacy, Tongji Hospital, Tongji Medical College, Huazhong University of Science and Technology, Wuhan, 430030 China

**Keywords:** Organ preservation solution, Low-viscosity, Protect, Kidney, Graft

## Abstract

Organ preservation solutions are designed to minimize organ damage during transplantation. A novel preservation solution, WMO-II, was developed to have a low viscosity and to improve microvasculature perfusion for kidneys. In an autologous canine transplantation model, kidney function and recovery were evaluated after organs were flushed and cold-stored with WMO-II or HTK solution, a perfusate currently approved for clinical use. The average number of red blood cells remaining in a single glomerulus after flushing with WMO-II was significantly reduced when compared with HTK solution. Additionally, WMO-II reduced the number of apoptotic bodies in stored kidneys compared to HTK treated tissue after 48 h of cold storage by reducing expression of Caspase-9, BiP, Chop, and Caspase-12. WMO-II solution reduced serum creatinine levels and serum potassium in kidneys stored for 48 h when compared to HTK perfusion. WMO-II preserves kidney function as evidenced by the reduction in serum creatinine and potassium during graft transplantation.

**Electronic supplementary material:**

The online version of this article (10.1186/s13578-018-0226-2) contains supplementary material, which is available to authorized users.

Dear Editor,

Transplantation has become the most effective treatment for patients suffering end-stage organ failure. During transplantation, tissue grafts suffer multiple stresses such as warm and cold ischemia, which are detrimental to early graft function and result in delayed graft function (DGF) or even primary non-function (PNF) [1]. To preserve early graft function, a preservation solution is required for the thorough flushing and cold storage of organs to prevent cell swelling and contracture due to energy loss and ameliorating free radical-induced injury.

The severity of ischemic damage is directly correlated with the extent of microcirculatory reperfusion failure [2]. To reduce harm to kidneys from recipient immune reaction, a low-viscosity flush solution may effectively wash out donor blood cells, inflammatory mediators, and vasoactive substances [3]. Over the past several decades, HTK and UW solutions are the most commonly used and are considered effective and safe [4, 5]. Unfortunately, ischemia injury-induced renal damage with resultant DGF remains relatively high, ranging from 20 to 40% in transplant recipients [6], suggesting the need for optimizing current available solutions. In addition to a need for optimizing solutions, there is a push to expand the criteria for donors to increase the number of organs available for transplantation. This indicates that there is still a need for optimizing perfusion solutions to reduce the ischemia-induced renal injury.

Here, we have developed a novel, low-viscosity solution, Wuhan Medical College organ preservation solution-II (WMO-II). In this study, the WMO-II solution was characterized and compared to another low-viscosity preservation solution, HTK. Kidneys were evaluated in an autologous canine transplantation model to assess efficacy on microvasculature perfusion and function without immune interference in both in vivo and ex vivo models.

## Results and discussion

The WMO-II solution we describe was designed by the Institute of Organ Transplantation and the Pharmacy Department of Tongji Hospital, Tongji Medical College, Huazhong University of Science and Technology (Table 1). The product consists of two connected inner bags and an over pouch. The inner bags are separated into Chamber A and Chamber B by a peel-able seal that can be opened with a moderate squeeze to mix solutions A and B (Additional file 1: Figure S1). An oxygen absorber is placed between the inner bag and the over pouch. Based on the components of solutions A and B, the WMO-II solution remains stable when stored at room temperature or 4 °C. Before use, the solution is mixed and cooled to 4 °C.

**Table 1:** Composition of WMO-II solution

Chamber	Component	Concentration (mmol/L)	Volume (mL)
A	Potassium citrate	23.4–28.6	500
	Sodiumdi hydrogen phosphate	3.6–4.4	
	Disodium hydrogen phosphate	22.5–27.5	
	Magnesium sulfate	4.5–5.5	
	Potassium chloride	1.8–2.2	
	Mannitol	143–158	
	Adenosine	4.5–5.5	
	Acetyl cysteine	4.3–5.8	
	Sodium hydroxide	4.3–5.8	
	Water for injection		
B	Dextran 40	–	500
	Water for injection		

**Fig. 1:**
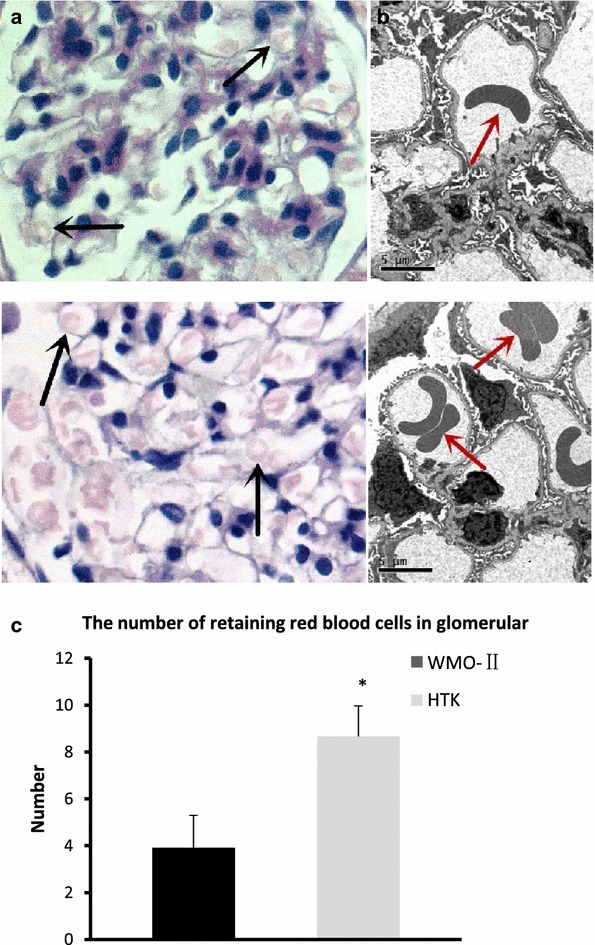
Perfusion with WMO-II improves clearance of red blood cells in kidneys. **a** Representative images of H&E staining of ex vivo canine kidneys flushed with 150 mL either solution at 4 °C via the artery for 5 min or until the effluent ran clear. Black arrows: retained red blood cells. **b** Representative TEM images of a single glomeruli of ex vivo canine kidneys after perfusion with WMO-II or HTK solutions. Red arrows: retained red blood cells. **c** The average number of blood red cells retained in a single glomerulus after perfusion with WMO-II or HTK solutions. Each kidney was prepared in 2 sections (1 cm × 1 cm) and all glomeruli in each section were examined. Data expressed as mean with standard error. n = 6 *P < 0.05

To assess the potential for the WMO-II solution, we first conducted experiments with ex vivo kidneys derived from dogs. We perfused kidneys with either the WMO-II solution or HTK, another low viscosity solution, and assessed clearance of red blood cells from the glomeruli and microvasculature of the kidneys. The H&E stain in Fig. 1a shows WMO-II greatly reduced the number of red blood cells retained in a single glomerulus compared to HTK. Additionally, the TEM images in Fig. 1b clearly show WMO-II perfused kidneys retained fewer red blood cells in the microvasculature than HTK perfused kidneys. On average, fewer red blood cells were observed in a single glomerulus perfused with WMO-II solution (3.9 ± 1.4) compared with 8.7 ± 1.3 in the HTK group (*P *< 0.05) (Fig. 1c), consistent with our theory that a lower viscosity solution would be more effective at clearing the microvasculature of the kidney. This is particularly important as red blood cells, when lysed due to changes in osmotic pressure or other signals of cell death, release many oxidative species, damaging surrounding tissue. Additionally, complete perfusion of microvasculature can help reduce immunological side effects, an aspect of WMO-II that will be assessed in future studies.

**Fig. 2:**
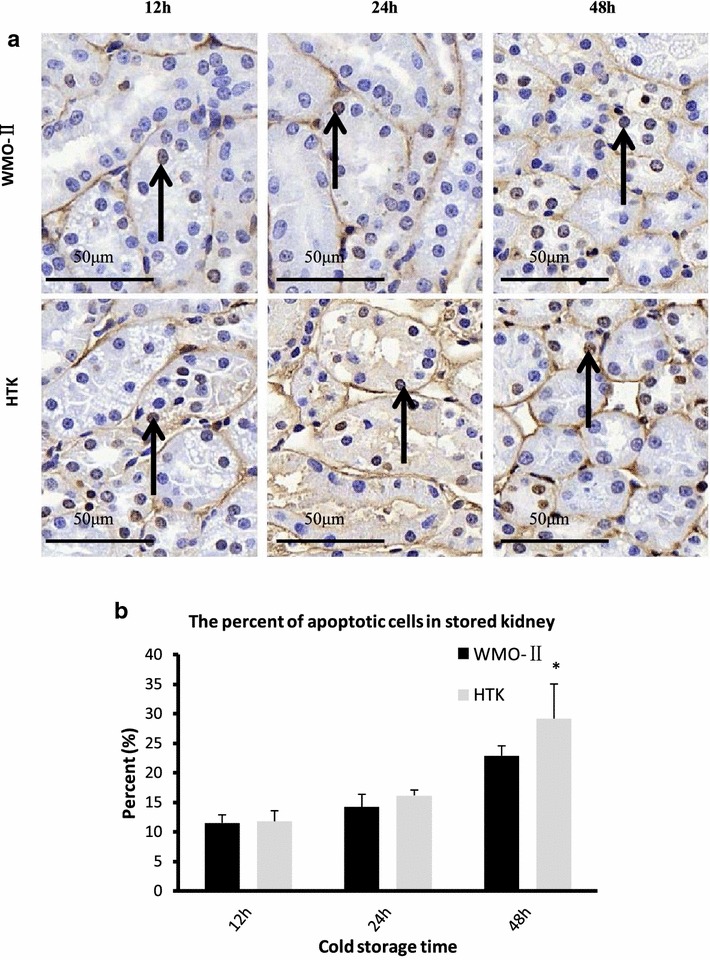
WMO-II reduces apoptosis in ex vivo kidneys after cold storage. **a** Representative TUNEL images of kidneys perfused and stored with WMO-II or HTK solutions and stored at 12, 24, or 48 h at 4 °C. Black arrows indicate cells with DNA fragmentation. **b** Quantification of TUNEL assay. Data expressed as mean with standard error. n = 6 *P < 0.05

**Fig. 3:**
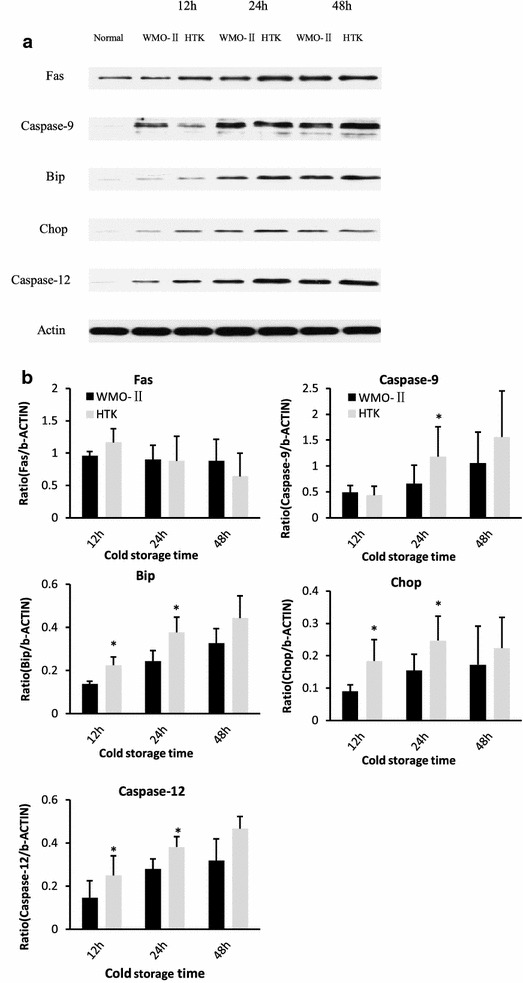
WMO-II prevents apoptosis by down-regulating mitochondrial and ERS-mediated apoptotic signaling. **a** Representative western blot images of Fas, Caspase-9, BiP, CHOP, Caspase-12, and β-Actin from tissues perfused and stored with WMO-II or HTK solutions for 12, 24, or 48 h. **b** Western blot signal quantification via AlphaEaseFC™ software and normalized to β-actin signal. Data expressed as mean with standard error. n = 6 *P < 0.05

Donated kidneys often undergo a relatively long cold ischemia time (CIT) because kidneys are often transported long distances. If kidneys can stay functional under prolonged CIT, the pool of donors would greatly expand. We designed the WMO-II solution to incorporate more antioxidants to reduce the amount of cellular damage caused by the oxidative stress associated with CIT. In comparison with HTK, WMO-II does not use histidine, due to histidine’s propensity for enhancing the formation of reactive oxygen species for cell injury [7] and augments mannitol since it removes reactive oxygen species as a sugar alcohol. Additionally, WMO-II contains adenosine, acetylcysteine and dextran for prevention of free radicals. Adenosine prevents tissue damage during instances of hypoxia and ischemia [8]; acetylcysteine is a medication that is used to loosen thick mucus and helps reduce viscosity of the WMO-II solution [9]; and dextran is a complex branched glucan used to reduce blood viscosity, decreasing vascular thrombosis by reducing erythrocyte aggregation, and decreasing platelet adhesiveness [10]. These components result in less apoptosis by decreasing mitochondrial and ER stress. Imaging indicated only a few apoptotic tubular cells in kidneys treated with either WMO-II or HTK solutions for 12 and 24 h (Fig. 2a). The number of apoptotic tubular cells increased greatly in both the WMO-II (48 h) group and HTK (48 h) group. Excitingly, at 48 h, WMO-II perfusion reduced the amount of apoptotic tubular cells when compared to the HTK perfusion (22.87 ± 1.52 vs. 29.17 ± 5.34, *P *< 0.05) (Fig. 2b). The Fas-mediated (Fas), mitochondrial-mediated (Caspase-9), and ERS-mediated (BiP, Chop, and Caspase-12) pathways were examined using western blot analysis (Fig. 3a). Normal kidney tissue was compared with kidneys stored in the WMO-II or HTK solutions for 12, 24 and 48 h. As shown in Fig. 3b, there were, on average, no significant differences in the expression of Fas between the WMO-II and HTK groups. However, expression of Caspase-9 was lower (*P *< 0.05) in the WMO-II (24 h) group than in the HTK (24 h) group. Moreover, expression of BiP, Chop, and Caspase-12 were lower (*P *< 0.05) in the WMO-II (12 and 24 h) group than their counterparts in the HTK (12 and 24 h) group. Although causation of a reduction in red blood cells causing decrease in in damage is not proven in this study, there was a significant reduction in apoptotic cell bodies in kidneys treated with WMO-II solution with a concurrent decrease in mitochondrial and ERS-mediated apoptosis markers which could be attributed to either a reduction in red blood cell retention or inhibition of oxidative species with the anti-oxidant properties of the solution.

In addition to assessing cell viability after CIT, a new perfusate would be useless if it did not also improve tissue function after transplantation. An auto-transplantation model was used to test the renal protective effect of the WMO-II solution in an antigen-independent model. Animals were randomly assigned to groups designated by preservation solution (WMO-II and HTK) and cold storage time [12 h (n = 5), 24 h (n = 10), and 48 h (n = 10)]. The recipient animals were monitored for up to 10 days to assess kidney function by serum creatinine (SCr) and potassium (K^+^) concentrations.

**Fig. 4:**
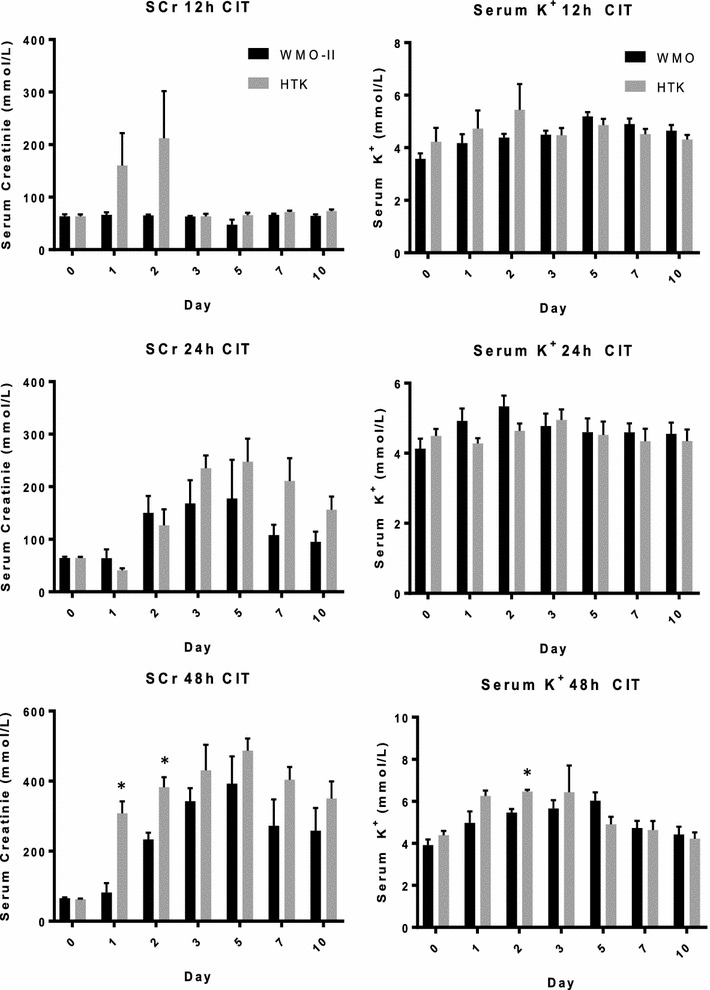
WMO-II maintains kidney function after cold storage 10-days after auto-transplantation. Concentration of serum creatinine (SCr) and serum potassium from blood samples collected daily from dogs auto-transplanted with WMO-II or HTK cold-stored kidneys stored for either 12, 24, or 48 h at 4 °C. Data expressed as mean with standard error. (Top) 12 h n = 5, (middle) 24 h n = 10, (bottom) 48 h n = 7 *P < 0.05

In the first group, the kidneys were stored for 12 h, resulting in minimum damage to the kidney, as shown in Fig. 4 (top), and a trend is seen where storage in WMO-II solution has lower SCr and potassium than storage in HTK solution. This trend becomes clear at 24-h storage (Fig. 4, middle), where sustained elevation of SCr is observe in HTK storage, whereas storage in WMO-II is lower. Finally, and most importantly, prolonged storage of the ex vivo kidneys for 48 h results in clear difference between the two organ preservation solutions. WMO-II significantly reduces SCr and serum potassium compared to HTK solution, indicating that WMO-II solution may be improving CIT time over previous preservation solutions. Since we have a limited number of dogs used in the present study, other days or storage times did not reach significance.

This study showed that WMO-II is more effective at clearing red blood cells from the microvasculature of perfused kidneys. Although the auto transplantation model used in this study is excellent for assessing the efficacy of perfusion and maintenance of kidney heath, it does not allow for the assessment of graft rejection or tissue damage caused by an immune response. We believe that by improving the clearance of the microvasculature, there will also be a reduction in white blood cells from the donor, reducing damage caused by the recipient’s immune system to result in a reduction of DFG and PNF, however, this must be tested in future experiments. Although the WMO-II solution is a clear improvement upon perfusion solutions that are currently standard in the clinical setting, we also look to investigate its effectiveness in prolonging warm ischemia time in the future, potentially reducing the damage caused by placing tissues on ice and further improving tissue survival and function for kidney transplantation.

## Materials and methods

### WMO-II solution

The WMO-II solution was designed by the Institute of Organ Transplantation and the Pharmacy Department of Tongji Hospital, Tongji Medical College, Huazhong University of Science and Technology (Table 1). The solution used for this study was manufactured by Huaren Pharmaceutical Co. Ltd., Qingdao, China, with our permission. The product consists of two connected inner bags and an over pouch. The inner bags are separated into Chamber A and Chamber B by a peel-able seal that can be opened with a moderate squeeze to mix solutions A and B (Additional file 1: Figure S1). An oxygen absorber is placed between the inner bag and the over pouch. Based on the components of solutions A and B, the WMO-II solution remains stable when stored at room temperature or 4 °C. Before use, the solution is mixed and cooled to 4 °C.

### Animals

All procedures involving animals in this study were performed in accordance with the guidelines for animal experiments of the Chinese Council on Animal Care, and were approved by the Institutional Animal Care and Use Committee of the Huazhong University of Science and Technology. The adult male beagle dogs were purchased from the Experimental Animal Center of Tongji Medical College. The animals, weighing 10.0 ± 1.8 kg (mean ± SD), were housed in metabolic cages and allowed to acclimatize to their surroundings for 1 week before the surgery. All animals were fasted 12 h prior to the surgery.

### Experimental design

An auto-transplantation model was used to test the renal protective effect of the WMO-II solution. The HTK solution was used as a control solution because it is another low-viscosity perfusate that is already approved for clinical use. The animals were randomly assigned to groups designated by preservation solution (WMO-II and HTK) and cold storage time [12 h (n = 5), 24 h (n = 10), and 48 h (n = 10)]. The recipient animals were monitored for up to 10 days to assess kidney function by serum creatinine (SCr) and potassium (K^+^) concentrations.

Another series of experiments with animals analyzed the protective mechanisms of the WMO-II. The left and right kidneys were removed for flushing and ex vivo storage (left by WMO-II, and right by HTK). After kidney procurement, the donors were euthanized. The kidneys of each animal were designated into two groups: WMO-II (0, 12, 24 and 48 h) groups and HTK (0, 12, 24 and 48 h) groups (n = 6 kidneys per group). At the designed time-points of cold storage, the kidneys were prepared for glomerular microvasculature perfusion analysis, pathological evaluation, transmission electron microscopic analysis, and apoptotic analysis.

### Kidney procurement and auto-transplantation

The canine auto-transplantation model is already established. Briefly, animals were heparinized with 50 U/kg of heparin, and the left kidney was collected as the donor kidney by cutting the ureter at the iliac vessel and transecting the renal artery and vein from the aorta and vena cava. Kidneys were flushed with 150 mL of the WMO-II solution or the HTK solution at 4 °C via the artery for 5 min or until the effluent ran clear. The kidney was stored in cold solution at 4 °C for 12, 24, or 48 h. After surgery, the animals were given Ringer’s solution supplemented with 10% glucose as well as food and water up to 12 h before the auto-transplantation surgery.

For auto-transplantation, the animals were re-anesthetized at the time corresponding to the experimental cold storage time. The kidney artery was anastomosed end-to-end to the external iliac artery, and the kidney vein was anastomosed end-to-side to the external iliac vein. After ureterocystostomy, right nephrectomy was performed to examine only the effects of the transplanted kidney. At 10 days post operation, animals were intravenously euthanized with an overdose of pentobarbital (100 mg/kg) and the auto-grafts were collected for further analysis.

### Glomerular microvasculature perfusion analysis

Six kidneys from the WMO-II (0 h) group and HTK (0 h) group were analyzed. Each kidney was prepared in two sections (1 cm × 1 cm) for hematoxylin and eosin (H&E) staining. All glomeruli in each section were evaluated under 200× magnification and the average number of red blood cells retained in a single glomerulus in each group was counted.

### Transmission electron microscopic analysis

Tissues were immediately immersed in 2.5% glutaraldehyde for 2 h. After being washed three times in PBS (pH 7.4), the tissue was post fixed in 1% osmium tetroxide for 30 min, dehydrated through graded alcohol and acetone, and embedded in Epon 812. Ultrathin sections were stained with uranyl acetate and lead citrate and imaged with a Philips CM10 transmission electron microscope (FEI, Philips, Eindhoven, Netherlands).

### Apoptosis analysis

Apoptosis was detected by terminal deoxynucleotidyl transferase-mediated dUTP-biotin nick end labeling (TUNEL) assay with ApopTag technology (Millipore, Billerica, MA, USA) according to the manufacturer’s instructions. Apoptosis was quantified by counting the number of positive cells per 200× field.

### Western blot analysis

Kidney tissue was homogenized in 1× RIPA buffer (Millipore, Billerica, MA) with 2% Halt Protease Inhibitors and 1% Halt Phosphatase Inhibitors (Pierce Biotechnology, Rockford, IL, USA). The lysates were spun down at 11,000 g for 15 min and the protein concentrations of the supernatant were measured using the Bradford Method (Bio-Rad, Hercules, CA). Protein expression of Fas-mediated (Fas, dilution, 1:1000, BD Biosciences San Jose, CA), mitochondrion-mediated (Caspase-9, dilution, 1:1000, BD Biosciences San Jose, CA) and endoplasmic reticulum stress (ERS)-mediated (BiP, dilution, 1:1000; Chop, dilution, 1:2000 and Caspase-12, dilution, 1:2000) (BD Biosciences San Jose, CA) apoptotic pathways were examined. Band densities were determined using AlphaEaseFC™ (Alpha Innotech) software and normalized to corresponding β-actin signal.

### Kidney function evaluation

Blood samples were drawn before the surgery and every 24 h post-transplantation. Samples were evaluated for serum levels of SCr and potassium (K^+^). The specific procedures were according to the kits protocol (Sigma Diagnostics Co. St. Louis, MO).

### Statistical analysis

Statistical analysis was performed using SPSS (version 17.0; SPSS Inc., Chicago, IL, USA). Quantitative data were presented as mean ± SEM. Statistical differences between groups at each time point were tested by three-way or two-way analysis of variance (ANOVA), followed by a Bonferroni correction. Mann–Whitney *U*-test was used for non-normally distributed data, and unpaired two-tailed Student’s *t*-test was performed for normally distributed data. *P *< 0.05 was considered statistically significant.

## Additional file

**Additional file 1: Figure S1.** WMO-II solution: The product consists of chamber A and chamber B separated by a peelable seal that can be opened with a moderate squeeze to mix for use.

## Abbreviations

DGF: delayed graft function
PNF: primary nonfunction
UW: University of Wisconsin
HTK: histidine–tryptophan–ketoglutarate
WMO-II: Wuhan Medical College organ preservation solution-II
cP: centi-Poise
SCr: serum creatinine
CIT: cold ischemia time
