# Correction to: A novel organ preservation solution with efficient clearance of red blood cells improves kidney transplantation in a canine model

**DOI:** 10.1186/s13578-020-00421-3

**Published:** 2020-05-09

**Authors:** Sheng Wang, Kristyn Gumpper, Tao Tan, Xianzhang Luo, Hui Guo, Changsheng Ming, Hanying Jiang, Jiangguo Fang, Guang Du, Hua Zhu, Jianjie Ma, Zhishui Chen, Nianqiao Gong

**Affiliations:** 1 Institute of Organ Transplantation, Key Laboratory of the Ministry of Health and the Ministry of Education, Tongji Hospital, Tongji Medical College, Huazhong University of Science and Technology, Wuhan, 430030 China; 2 Department of Surgery, Davis Heart and Lung Research Institute, The Ohio State University, Columbus, OH 43210 USA; 3 Department of Molecular and Cellular Biochemistry, The Ohio State University, Columbus, OH 43210 USA; 4 Department of Pharmacy, Tongji Hospital, Tongji Medical College, Huazhong University of Science and Technology, Wuhan, 430030 China

## Correction to: Cell Biosci (2018) 8:28 10.1186/s13578-018-0226-2

Following publication of the original article [1], one of the funding was missing in the Funding section.

The updated funding is given below.

## Funding

This work was supported by Grants to NQ Gong from the National Natural Science Foundation of China (No. 81570678), to NQ Gong from the Clinical Research Physician Program of Tongji Medical College, HUST, to NQ Gong from Major State Basic Research Development Program of China (No. 2013CB530803, 973), to CS Ming from the National High-Tech Research and Development Program (Program 863) of the Ministry of Science and Technology of China (2012AA021010), and to ZS Chen from the Translational Grant of Tongji Hospital. JM acknowledges support from grant RO1DK106394.
