# Supplementary material for: A novel organ preservation solution with efficient clearance of red blood cells improves kidney transplantation in a canine model
Source: Cell Biosci. 2018 Apr 10;8:28. doi: 10.1186/s13578-018-0226-2 (PMC5894212; doi:10.1186/s13578-018-0226-2)

**Additional File**

Additional File 1: Figure 1. WMO-II solution**:** The product consists of chamber A and chamber B separated by a peelable seal that can be opened with a moderate squeeze to mix for use.


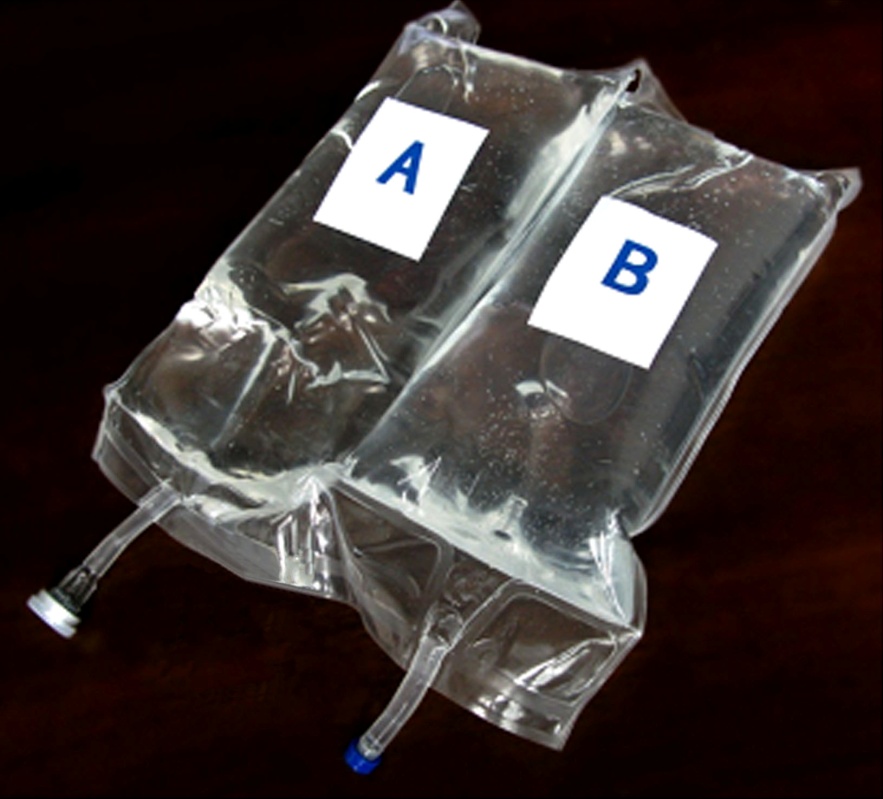

Supplement: Supplementary file 1 — Additional file 1: Figure S1. WMO-II solution: The product consists of chamber A and chamber B separated by a peelable seal that can be opened with a moderate squeeze to mix for use. [file 13578_2018_226_MOESM1_ESM.docx]
